# Managing Hospital Employees’ Burnout through Transformational Leadership: The Role of Resilience, Role Clarity, and Intrinsic Motivation

**DOI:** 10.3390/ijerph191710941

**Published:** 2022-09-01

**Authors:** Jinyong Chen, Wafa Ghardallou, Ubaldo Comite, Naveed Ahmad, Hyungseo Bobby Ryu, Antonio Ariza-Montes, Heesup Han

**Affiliations:** 1 Business School, Hubei University, Wuhan 430062, China; 2 Department of Accounting, College of Business Administration, Princess Nourah bint Abdulrahman University, P.O. Box 84428, Riyadh 11671, Saudi Arabia; 3 Department of Business Sciences, University Giustino Fortunato, 82100 Benevento, Italy; 4 Faculty of Management, Department of Management Sciences, Virtual University of Pakistan, Lahore 54000, Pakistan; 5 Faculty of Management Studies, University of Central Punjab, Lahore 54000, Pakistan; 6 Food Franchise Department, College of Health Sciences, Kyungnam University, 7 Kyungnamdaehak-ro, Masanhappo-gu, Changwon-si 51767, Korea; 7 Social Matters Research Group, Universidad Loyola Andalucía, C/Escritor Castilla Aguayo, 4, 14004 Córdoba, Spain; 8 College of Hospitality and Tourism Management, Sejong University, 98 Gunja-Dong, Gwanjin-gu, Seoul 143-747, Korea

**Keywords:** transformational leadership, medical errors, burnout, healthcare

## Abstract

Medical errors have been identified as one of the greatest evils in the field of healthcare, causing millions of patient deaths around the globe each year, especially in developing and poor countries. Globally, the social, economic, and personal impact of medical errors leads to a multi-trillion USD loss. Undoubtedly, medical errors are serious public health concerns in modern times, which could be mitigated by taking corrective measures. Different factors contribute to an increase in medical errors, including employees’ risk of burnout. Indeed, it was observed that hospital employees are more exposed to burnout situations compared to other fields. In this respect, managing hospital employees through transformational leadership (TL) may reduce the risk of burnout. However, surprisingly, studies on the relationship between TL and burnout are scarce in a healthcare system, indicating the existence of a critical knowledge gap. This study aims to fill this knowledge gap by investigating the role of TL in reducing the risk of burnout among hospital employees. At the same time, this study also tests the mediating effects of resilience and role clarity with the conditional indirect effect of intrinsic motivation in the above-proposed relationship. To test different hypotheses, a hypothetical model was developed for which we collected the data from different hospital employees (*n* = 398). Structural equation modeling (SEM) was considered for statistical validation of hypotheses confirming that TL significantly reduces burnout. The results further indicated that resilience and role clarity mediate this relationship significantly. Lastly, the conditional indirect effect of intrinsic motivation was also confirmed. Our results provide meaningful insights to the hospital administrators to combat burnout, a critical reason for medical errors in hospitals. Further, by incorporating the TL framework, a hospital may reduce the risk of burnout (and, hence, medical errors); on the one hand, such a leadership style also provides cost benefits (reduced medical errors improve cost efficiency). Other different theoretical and practical contributions are discussed in detail.

## 1. Introduction

According to a recent World Health Organization (WHO) report, around 2.6 million patients die each year due to medical errors, especially in developing and low-income countries [1]. The report further adds that most of these deaths could be avoided by taking corrective actions to improve medication errors. Globally, the social, economic, and personal impact of medical errors leads to a multi-trillion USD loss. Undoubtedly, medical errors are serious public health concerns in modern times. Most critical medical errors are related to poor diagnosis, use of medicines, improper patient handling, and prescription. More than USD 40 billion is associated with medical errors globally [2]. To make the picture bleaker, around 25% of patients’ complications are due to poor surgical care procedures, resulting in one million deaths yearly. Compared to developed countries in the world, developing countries present an alarming situation where more than 700 patients die each day due to different medical errors [3].

The above discussion is enough to highlight the critical issue of medical errors in a healthcare system. However, unfortunately, a universal solution to reduce the medical error rate in a healthcare system is not available. Even if such a solution has existed, it is challenging to provide a consistent solution to reduce the medical error rate [4]. Nonetheless, there is a general consensus among scholars that patient safety can be improved by learning from such events that lead to medical errors [5]. The observation is not new that a stressful working environment that most healthcare staff face is one of the critical causes that give rise to job burnout, which ultimately increases the medical error rate [6,7]. Theoretically speaking, burnout in an organizational context is a psychological response to workplace stress. Knowing the critical importance of job burnout, the scholarly interest in understanding the factors of job burnout has increased over the past two decades [8,9]. For example, it was specified that factors such as role conflict [10], role stress [11], role clarity [12], and work overload [13] could give rise to job burnout in an organizational context. Scholars have also investigated the factors that could mitigate the negative effect of burnout. Among different factors, perhaps, an effective leadership style, for example, transformational leadership style (TL), was reported as a significant organizational factor to reduce the level of burnout among the followers (employees in this case) [14,15]. A transformational leader is a person who is capable of nurturing the employees up from their preoccupations and leading them towards a common purpose [16]. An effective leadership style in an enterprise is essential to its success. Though most corporate leaders employ a primary style of enterprise management, differences in cultures and values are of seminal importance for a corporate leader. This implies that cross-cultural differences are to be expected in a leadership style. Extending this debate, Tara [17] mentioned that leadership in various cultures differs with respect to the use of power. For example, individuals who desire to increase their personal gains show individualist-related behavior, whereas people with a collectivist culture are inclined to help the community. The study conducted by House, et al. [18] in 62 societies indicated that cultural differences do influence global leadership. For instance, in Asian enterprises, political interventions and family control are likely to be more common. Conversely, in the USA, corporate leaders tend to groom their successors from talented employees. Although the role of TL in influencing employee outcomes was established in the prior literature, such studies in a healthcare context remained underexplored, with some exceptions [19,20]. However, the focus of such studies was either the developed countries, or developing countries close to being announced as developed (China, for example), leaving the terrain of developing countries unattended. Therefore, to bridge this knowledge gap, we aim to advance this debate on the relationship between TL and burnout in a healthcare context of a developing economy.

Avolio and colleagues [21] were among the first who highlighted the importance of certain psychological factors as mediators to explain the mechanism between TL and followers’ outcomes. Since then, many researchers have introduced different mediators in the relationship between different leadership styles and employees’ outcomes in an organizational context [12,20,22]. Among different psychological factors, the mediating effect of role clarity and resilience to reduce the negative outcomes of burnout was highlighted by different scholars [12,23,24]. Resilience is described as a psychological factor that an individual possesses to get rid of extreme situations they face in a workplace context [25]. Similarly, role clarity is defined as the extent to which employees perceive they have clear guidance pertinent to anticipated roles and behaviors for a job [26]. Despite the fact that the mediating effects of role clarity and resilience were mentioned in previous studies, such mediating effects in a healthcare context, from a TL perspective, were not investigated earlier. This indicates the need to explore the mediating effects of role clarity and resilience in the healthcare segment.

The literature also suggests that different individual factors also influence employees’ burnout perceptions. For example, the moderating roles of psychological capital [27] and locus of control [28] to buffer burnout perceptions of employees were highlighted previously. In this vein, recently, some scholars went to a great extent in establishing the moderating role of intrinsic motivation to reduce the negative effect of burnout on the part of employees [29,30]. Intrinsic motivation is defined as a personal characteristic of an individual to implement an action for inherent satisfaction instead of external reasons [31]. Interestingly, the relationship between TL and intrinsic motivation has been established by previous researchers [32,33]. However, the conditional indirect effect of intrinsic motivation between the mediated relationship of TL and burnout through role clarity and resilience was not discussed earlier. We argue here that intrinsic motivation can buffer employees’ burnout perceptions in a certain organizational context, and, as TL was reported to influence intrinsic motivation positively, it will be worthwhile to investigate this conditional indirect effect.

The target segment of this study is Pakistan’s healthcare sector, which is taken into consideration for the following reasons. First, according to a recent report, almost 700 individuals die on a daily basis due to medical errors in developing countries, including Pakistan [3]. As burnout was identified a critical factor that gives rise to medical errors in a healthcare system [34,35], it is critical to investigate the factors that can reduce the level of burnout among healthcare employees. Second, according to a recent report, burnout among healthcare employees has been increasing worldwide and emerging as a critical problem in this sector [36]; therefore, it is worthwhile to study the factors that can reduce the risk of burnout among healthcare employees. Third, compared to developed countries, the health systems in most developing countries are overburdened [37]. Pakistan, as a developing country, also faces the same situation. Healthcare employees have to face stressful situations daily, leading them towards burnout. In this respect, a recent nursing study shows that around 50% of nurses experience the risk of burnout [38], which is alarming. Therefore, it will be worthwhile to help this sector to deal with this critical issue by employing a TL approach.

## 2. Theory and Related Literature for Hypotheses Development

### 2.1. Theoretical Underpinning

This study uses the theoretical groundings of conservation of resources theory (CRT) to provide a theoretical justification for hypothesized relationships. Proposed by Hobfoll [39], this theory suggests that burnout is a consequence of resource loss. Resources may be regarded as anything that employees in an organizational setting think of as helpful in achieving different organizational goals and objectives [40]. In this regard, employees face stress when they perceive they have insufficient resources or they lose some necessary resources. Hobfoll further argued that burnout occurs on the part of employees when they feel their resources are lost. To recover from such a situation, employees need more resources to be invested in them [41]. To this end, the role of corporate leaders is very important, because an affective corporate leader provides employees with different valuable resources, for instance, providing them with the needed feedback, support, praise, and guidance [42]. Previous researchers have also used this theory to support their theoretical arguments on employee burnout in different organizational contexts [43,44]. Even some scholars in recent studies employed this theory in a leadership framework [45]. We, therefore, feel it relevant to consider this theory for theoretical justification of the current work.

### 2.2. Hypotheses Development

Generally, the literature discusses burnout on three major dimensions, including emotional exhaustion, depersonalization, and reduced personal accomplishment [46,47]. With this respect, it was argued that corporate leaders are in a great position to influence different employees’ outcomes, including emotions and motivation. It was also specified in the prior literature that leaders provide resources to their followers, which ultimately reduces the risk of burnout [48,49]. The existing literature has discussed different leadership styles to reduce employee burnout in a healthcare context. Among different leadership styles, the literature suggests that an authentic leader reduces healthcare employees’ burnout by empowering, optimizing, and enhancing their job autonomy [50]. LuthansandAvolio [51] defined authentic leadership as a style of organizational management that focuses on fostering positive psychological capacities of the followers in a highly developed organizational context that enhances employees’ self-awareness and self-regulations. At the same time, an authentic leader positively affects employees’ key psychological processes of positive emotions, hope, and optimism [52], which then reduces their burnout. Scholars have also mentioned the importance of an ethical leadership style to reduce employee burnout in healthcare. Ethical leadership is described as the process in which a leader demonstrates a normatively appropriate behavior via personal acts, maintains interpersonal interactions with the followers, and involves them in decision-making processes [53]. An ethical leader focuses on employees’ well-being in an organization, tries to solve their problems, and develops a positive working environment [54]. When employees work in a positive working environment, it is likely to reduce different work-related stress, including burnout [55]. Similarly, recent literature related to healthcare also acknowledges the role of TL in reducing healthcare employees’ burnout. 

Specifically, the role of TL in reducing employees’ burnout in an organization was discussed at several points in the previous literature [14,15]. TL motivates and inspires the employees to put forth their best efforts to achieve organizational goals. Specifically, a corporate leader, such as TL, focuses on developing its employees by improving their capabilities, recognizing their efforts, and motivating them through appreciation [56]. At the same time, a TL shows respect, pride, and trust to the followers [57]. Jain, et al. [58] indicated that employees working under a corporate leader who follows the philosophy of TL feel trusted and respected, which urges them to put forth more efforts to achieve different organizational objectives. Generally, the literature establishes that, under the supervision of TL in an organization, employees face less risk of burnout [14,59,60]. In a healthcare context, employees are particularly exposed to situations that increase burnout risks. High patient-to-staff ratios, lack of social support, increased workload, and intense interaction with patients are some common norms in this sector, due to which employees feel a resource loss, giving rise to the risk of burnout among healthcare employees [19]. In this respect, TL can provide meaningful help to employees by providing them with added support, guidance, appreciation, etc. In essence, we theorize that TL as a contextual resource may extend the pool of resources for employees in an organization, which ultimately reduces the risk of burnout. Therefore:

**H1.**  *The presence of a transformational leader in an organization reduces job burnout in employees.*

Studies have shown that several organizational factors drive burnout in an organizational context. The work by CordesandDougherty [61] suggests three categories that give rise to the risk of burnout: job and role characteristics, organizational characteristics, and personal characteristics. Maslach, et al. [62] further added that the first two categories are more critical reasons (situational factors) for burnout compared to the third one. Examples of different situational factors include job characteristics, role clarity, and role overload. From a leadership perspective, role clarity implies that employees in an organization are fully aware of their responsibilities, tasks, and roles in an organization or group. Further, the employees do understand what their leadership expects from them [63]. A leader’s behavior in an organization, thus, serves as a key driver of experienced role clarity. Indeed, role clarity directly provides a better understanding of the job to the employees, which increase their performance [64]. Referring to the demand-control model proposed by Karasek [65], we argue that a better job understanding (role clarity) should lead employees towards a less stressful condition, which reduces burnout. This argument is also asserted by a number of prior researchers [66,67]. Conversely, the lack of role clarity leads employees to risk of stress and anxiety [68]. To this end, TL provides a clear role for employees, as such leaders provide proper guidance to employees for their jobs. Additionally, since TL treats employees with respect and shows concern for them, the work is formally and fairly distributed among all employees, implying that everyone knows his or her role clearly (role clarity). In line with the prior work [69,70,71], we expect that a corporate leader with transformational orientation will provide sufficient role clarity to the employees under his/her supervision and will help and guide them appropriately. Thus, role clarity not only reduces burnout directly, but also mediates the relationship between TL and burnout. Therefore:

**H2.**  *Role clarity reduces the risk of burnout in an organization.*

**H3.**  *Role clarity mediates the negative relationship between TL and burnout.*

Resilience constitutes one of the leading psychological factors related to employees’ emotional well-being and professional success [72]. The early researchers conceptualized resilience as the ability of an individual to adapt or cope with extreme working situations and life stressors [73,74,75]. However, scholars such as Richardson [76] and Thies and Travers [77] have argued that resilience is a dynamic quality of an individual that varies between individuals with respect to the extreme situations they face. King [78] contends that resilience allows individuals to find and use different resources (internal and external) to overcome the negative effects of some adverse situations and regain equilibrium.

Research on resilience has presented two types of resilience, which include type-I resilience (focusing on ill-being) and type-II resilience (focusing on well-being) from the perspective of stressors. Whereas type-I resilience focuses on sustained, regained, and/or decreased levels of ill-being, for instance, less ill-being than expected, type-II resilience focuses on sustained, regained, and/or increased well-being levels (increased well-being than expectation) with respect to stressors or adversities [79]. In this aspect, some resilience scholars in the past have focused on well-being (type-II), for example, life satisfaction and other positive effects [80,81], whereas others have focused on mental health problems, such as depression, anxiety, etc. [82,83]. To this end, we are in line with Huppert [84], who suggested that mental ill-being and well-being are the opposite poles of the same scale. Nevertheless, mental ill-being and well-being may represent separate but related unipolar dimensions [79]. Some studies have also suggested that mental ill-being and well-being have a moderate level of phenotypic overlap [84,85] and are influenced by both genetics and environmental sources [86,87,88]. Alternatively, the existence of well-being does not constitute a necessary condition for the absence of ill-being. In other words, genetic and environmental factors that influence well-being are not likely to be the same as those that contribute to less ill-being.

Scholars have studied resilience in stressors or adverse life situations [89]. Recently, resilience received increasing attention from academicians with respect to employees and workplace contexts [90,91]. The changing work dynamics, pressure situations in most organizations, and the blurred lines between the professional and personal life of employees are some of the critical factors that lead contemporary researchers to study resilience in order to deal with such situations [92]. Shin, et al. [93] proposed resilience as a mechanism for adjusting to a dynamic and uncertain working life. DiCorcia and Tronick [89] believed that resilience is a process of regulating workplace stressors. Indeed, resilience allows individuals to adapt to workplace fluctuations via flexibility of thoughts and actions [94]. At the same time, resilience enables an employee to adaptively gather, choose, or use different resources to react to different workplace stressors. Thus, resilience can reduce the risk of burnout among employees in an organization. This argument is also asserted by previous researchers [95,96].

Healthcare employees face stressful situations to which they have to adapt, react, or overcome [97]. Resilient employees can adapt and overcome adverse workplace situations [98]. Nevertheless, some scholars have argued that resilience can be built and influenced by different situations and contexts [99,100,101]. In this respect, the role of TL in a healthcare context is very important to enhance resilience among healthcare employees. The actions taken by an effective leader in response to different environmental challenges play a key role in affecting employee resilience [102]. An effective corporate leader can view problems as opportunities and takes different measures to convert crises into developmental opportunities [103]. Moreover, an effective leader increases employees’ psychological safety, maintains open communication with them, focuses on their growth and development, and builds trust [104]. All these factors eventually contribute to enhancing employee resilience. A leader with transformational orientation not only helps employees to reduce the risk of burnout, but he/she also executes strategies for the renewal of employees, including mindfulness practices and personal resilience plans [105]. Specifically, TL builds interpersonal relationships with the followers, which give birth to a healthier workplace environment, leading employees to a higher level of resilience [106]. The role of TL is critical for healthcare employees to motivate them to apply different conflict management tactics, develop highly resilient personnel, and reduce burnout. Additionally, TL, as a bottom-up approach [107], may influence employee resilience, which then reduces burnout. Thus, it can be theorized:

**H4.**  *Resilience reduces the risk of burnout in an organization.*

**H5.**  *Resilience mediates the negative relationship between TL and burnout.*

In the presence of intrinsic motivation, an individual is expected to commit an act for his/her passion rather than for external reward [108]. Theoretically, intrinsic motivation discusses three main characteristics of an individual, including a desire to be successful, the meaning of action, and orientation for personal life [109]. Indeed, employees with a higher level of intrinsic motivation are expected to focus on achieving their job tasks. Because such employees are focused and attentive, they put forth more effort to achieve the goals that their organization designates them [110]. Shin and Grant [111] believed that intrinsically motivated individuals in an organization see their job as interesting and accept workplace challenges optimistically. Moreover, such individuals have a higher level of determination as they gain fulfillment and a sense of accomplishment by successfully completing their job tasks. Vallerand [112] argued that employees with high intrinsic motivation level, have an increased level of vitality, absorption, self-esteem, and persistence. He further asserted, when intrinsically motivated employees face the risk of burnout, they have more personal resources to cope with such a negative situation. 

Knowing the potential benefits of intrinsic motivation, scholarly discussion has increased the understanding of how intrinsic motivation in an organizational context can be fostered [113]. Improving intrinsic motivation among employees leads not only an organization towards success, but also helps corporate leaders in saving time and money, compared to promoting extrinsic motivation [114]. In this respect, the seminal role of TL in promoting employees at a higher level of intrinsic motivation was highlighted by previous scholars [33,115]. A corporate leader under the philosophy of transformational leadership shares vision, mission, and trust with the followers on the one hand. He/she also inspires them to achieve organizational goals effectively, on the other hand. Moreover, TL shows a higher level of competence and determination in solving followers’ problems in the workplace, which ultimately increases their pride and dignity [116]. This whole process leads employees to the point where they are motivated and desire to exceed the designated tasks [117].

Additionally, the inspirational ability of TL focuses on enhancing the motivation level of employees by showing concern for fulfilling the needs and solving problems of employees. Altogether, TL in an organization not only provides role clarity to the employees, but also promotes intrinsic motivation among employees. Employees with a clear role in an organization and a higher level of intrinsic motivation face less stress and exhaustion, which eventually reduces the risk of burnout. 

Similarly, some recent surveys have indicated that motivation, especially intrinsic motivation, can influence resilience positively. In this respect, the work by Ghasem and Hosseinchari [118], León-Guereño, et al. [119], and Paul, et al. [120] can be mentioned as a few relevant examples. Specifically, the study by Mostafa and Lim [121] showed that the individuals who scored high in intrinsic motivation could hold a higher level of resilience. They further indicated that intrinsically motivated individuals are expected to commit more to their inherent satisfaction and professional aspiration. Moreover, such individuals are psychologically equipped with resilience. In this respect, as specified earlier in this draft, resilience can be learnt by the social processes and contextual factors, indicating that there is a role of leadership to influence employee resilience. In this aspect, the role of TL to boost the resilience of employees was already identified. Zhu, et al. [122] suggested that an effective leader, as a bottom-up approach, induces the intrinsic motivation of employees, which then triggers employee resilience. We argue that employees’ intrinsic motivation as an outcome of leadership may activate employee resilience. In addition, a transformational leader displays an objective self-evaluation, appreciation to employees for their achievements, and maintains open communications with them. Moreover, TL focuses on employees’ welfare, which enhances their intrinsic motivation level, which ultimately buffers their resilience and reduces employee burnout. Further, to enhance employees’ intrinsic motivation, a corporate leader helps followers understand their job’s meaning and purpose more effectively [123]. Therefore, in the existence of an effective leader, an organization tend to enhance the intrinsic motivation level of employees, which then buffers the mediated relationship between TL and burnout via role clarity and resilience. Thus, we propose the following hypotheses:

**H6.**  *Intrinsic motivation moderates the mediated relationship between TL and burnout through role clarity such that that the employees’ burnout is reduced when they have a higher level of intrinsic motivation.*

**H7.**  *Intrinsic motivation moderates the mediated relationship between TL and burnout through resilience such that that the employees’ burnout is reduced when they have a higher level of intrinsic motivation.*

**Figure 1:**
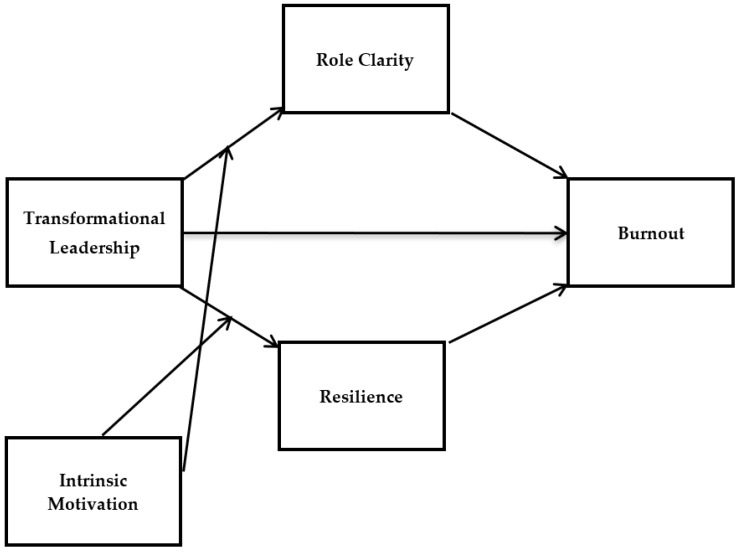
The hypothesized research model.

The proposed research model of this study is given in Figure 1 below.

## 3. Methodology

### 3.1. Unit of Analysis, Sample, and Procedure

We targeted the hospitals in the cities of Lahore and Karachi in Pakistan. Both cities constitute a multi-million population, and hospitals in Lahore and Karachi attend to a large and diverse umbrella of patients (both in-patients and out-patients). Moreover, not only are the people in these cities reliant on public and private health facilities in these two cities, but many patients from other parts of the country also visit different facilities in Lahore and Karachi. Currently, more than 13 million people live in Lahore, and more than 16 million people live in Karachi [124]. The fast-paced rising population in the country is also a reason to overburden the hospitals in Pakistan [125]. The hospitals in Pakistan are being administered by both the government and the private sector. However, most of the population is attended to by private hospitals [126]. Being included in the list of lower-middle-income countries, Pakistan’s healthcare system has been struggling regarding service delivery quality, performance, doctor–patient and nurse–patient ratios, infrastructure, and several other areas. Indeed, the country is at 154th place in health facilities in the list of 195 countries [127]. Medication error is one of the critical challenges faced by the healthcare system in Pakistan. Around half a million people die each year due to medical errors, which, if managed appropriately, could be avoided [128]. 

For the purpose of the data collection, different hospitals (both public and private) were approached with a request to co-operate in this survey activity for the larger interest of academia and the field. Seven hospitals (five from Lahore and two from Karachi) agreed to grant access to their employees for the data collection. Hence, the unit of analysis of this survey was individual employees serving in different hospitals. Specifically, the data collection activity was completed within a three-month time period (July to September 2021).

### 3.2. Instrument

We employed a questionnaire as a data-collecting instrument. This questionnaire was adapted from different sources for which detailed information is provided in the subsequent paragraphs. Indeed, the initial questionnaire version was presented to the experts [129,130,131,132]. The outlay of the questionnaire was comprised of three sections. The first page included the information regarding “the informed consent.” This page was provided to each respondent to fulfill one of the major ethical requirements given in the Helsinki Declaration [133,134,135]. In the second section, we requested the informants to share their socio-demographic information (age, gender, experience, etc.). Lastly, in the third section of the questionnaire, we invited the informants to rate their variables-related responses on a five-point Likert scale. This survey sample included employees and supervisors/leaders in different departments. We directly approached the employees of the selected hospitals for this data collection activity. Further, we employed a three-wave (separate) strategy for the data collection. An approximate time interval of two weeks was given in each wave. The socio-demographic information, intrinsic motivation (IMO), and burnout (BO)-related information were taken in one wave. Employees’ perceptions of their supervisor/leader (TL) were taken in another separate wave. The data related to resilience (RSL) and role clarity (RCL) were collected together in a separate wave. 

We measured the variables of this study by adapting the items from already published and reliable sources. For instance, the items of TL were adapted from Carless, et al. [136], who created a short version of TL called “Global transformational leadership scale” as a single construct. This scale consisted of seven items (for example: our leader gives encouragement and recognition to us). The original alpha value reported by the authors for this scale was 0.90. Similarly, BO was measured by using Copenhagen Burnout Inventory (CBI) scale developed by Kristensen, et al. [137]. They established a reasonable psychometric property of this scale by achieving a significant alpha value of 0.87. From this scale, we included seven items related to workplace BO (for example: I feel my work is emotionally exhausting). In a similar manner, the six statements to measure RLC were adapted from Rizzo, et al. [138] (for example: I have clear planned goals and objectives for my job). Brief Resilience Scale (BRS-6) developed by Smith, et al. [139] was used to measure RSL, which included six items (for example: I tend to bounce back quickly after hard times). The Cronbach’s alpha (α) in the original study ranged from 0.80 to 0.91 (in four samples). Lastly, five items to measure IMO were adapted from Tierney, et al. [140] (for example: I enjoy finding solutions to complex problems). The original α value reported by the authors for this scale was 0.74. The inter-item consistency calculated in this study was α = 0.876 for TL; 0.878 for BO; 0.869 for RLC; 0.862 for RSL; and 0.874 for IMO. Further, the adapted scales were publicly available for the readers. More detail on items is given in Appendix A.

### 3.3. Response Rate, Outliers, and Data Cleaning

We distributed 700 questionnaires initially, among which 247 were not returned back by the respondents. Specifically, in the first wave, we received back 510 filled questionnaires (almost 73%), whereas we received back 477 filled questionnaires in the second wave (68%). Finally, we received 453 filled questionnaires in the third wave (almost 65%). After data cleaning and detecting for outliers, 398 surveys were finally identified as useable. Hence, the overall response rate of this survey was around 57%. Because a sampling frame was unavailable, deciding on sample representativeness was not possible. Table 1 represents more information on the data cleaning process. In this respect, there were 37 responses that were not usable (missing data = 19 and outliers = 18). The contribution from female respondents was 43%, whereas 57% of respondents were male. The age of the respondents was divided into different groups (the first group was 18 years to 25 years and the last group was above 45 years). In this respect, most of the respondents were between the ages of 18 and 45 years (89%). The experience level of most respondents varied from 1 year to 7 years (the first group was 1 to 3 years and the last group includes employees with more than 10 years). More detail on socio-demographic information has been provided in Table 2. 

### 3.4. Reliability and Validity

The variables in this study were assessed for validity and reliability. At this stage, the convergent validity (CV) and composite reliability (CR) of all variables were tested. Usually, CV is a simultaneous measurement of the same construct by its items, and CR relates to inter-item consistency. The standardized factor loadings (SFL) of each item of a variable were taken into consideration to calculate CVs and CRs for all variables (TL, BO, RLC, RSL, and IMO). Detailed information on these values has been given in Table 3 below. The factor loadings of all items were significant (>0.7). Further, the factor loadings ranged from 0.707 to 0.819 for TL, from 0.701 to 0.833 for BO, from 0.700 to 0.863 for RLC, from 0.703 to 0.800 for RSL, and from 0.706 to 0.911 for IMO. It was observed that all CVs and CRs were significant because the average variance extracted (AVE) for all variables was above the cut-off value of 0.5 (establishing CV), and CRs were above 0.7 in all cases. This is in line with previous researchers [131,141,142].

## 4. Results

### 4.1. Model Fitness

To assess the model fitness, different measurement models (alternate) were developed in AMOS [143,144,145,146]. At the same time, we also developed the hypothesized five-factor measurement model. This five-factor model was assessed against different alternate models. To decide which measurement model best describes the theoretical model, we assessed different model fit indices (for example, normed fit index (NFI) and comparative fit index (CFI)), chi-square/degree of freedom, and root means square errors of approximation (RMSEA) were assessed. For more details on the obtained values against their acceptable ranges, we refer to Table 4. This activity led us to establish that only the theorized five-factor model showed superior model fit values in contrast to alternate measured models (NFI = 0.954, CFI = 0.952, *χ*^2^/*df* = 1.982, and RMSEA = 0.040).

### 4.2. Correlations

Correlations (*r*) between different variables of this study were also assessed to know the direction and intensity of the relationship between different variables. The output indicated mixed results (Table 5); for example, a positive *r* value amongst TL and RLC (T > RLC = 0.405) indicated both variables positively co-vary. Similarly, a negative *r* value amongst TL and BO established a negative association (TL <=> BO = −0.583). Nonetheless, no value showed an extreme case (*r ≥* 0.8), indicating that multicollinearity was not critical in this analysis. On a further note, we calculated discriminant validity (diagonal values in Table 5) and found that each value was greater than the value of *r*, implying that the items of one variable were different from others in all cases [147,148]. To explain further, it can be seen that the *r* values between TL-BO, TL–RLC, TL–RSL, and TL–IMO were all inferior to the diagonal value (0.778), which indicates that discriminant validity is significant. A similar observation can be seen in all other cases. 

### 4.3. Total, Direct, and Indirect Effects

We employed structural equation modeling (SEM) to test the hypothesized relationships [149,150,151]. For this purpose, we considered AMOS software. To draw the structural model, we followed the statistical model-7 guidelines given in PROCESS macro introduced by Hayes [152]. In this regard, based on the Hayes guidelines, we develop a user-defined syntax in AMOS to test the conditional indirect effect. Prior to testing for conditional indirect effect, the variables of TL and IMO were mean-centered in the SPSS data file. Moreover, the interaction term by multiplying TL with IMO was also developed for testing the moderating effect of IMO between TL and RLC, and between TL and RSL. We used 5000 bootstrapping samples to test mediating and conditional effects [153]. The output is given in Table 6. According to the results, TL negatively predicted (as was anticipated theoretically) BO, suggesting that H1 is accepted because both confidence interval (CI) values (lower and upper) did not include a zero point. Similarly, the effects of RLC and RSL on BO were also significant, supporting the theoretical statements of H2 and H4. Likewise, the indirect effects of RLC and RSL were also statistically significant, showing that, when included in the model as mediators, these variables further explained the negative relationship between TL and BO. These results were in favor of the statements of H3 and H5. Lastly, the conditional indirect effects of IMO on BO at different levels of moderator showed a statistical significance, implying that H6 and H7 should be accepted.

## 5. Discussion

Our results show that, in the presence of TL, employees feel less burnout risk in a hospital organization. A hospital manager, as a transformational leader, motivates and inspires the employees, on one side, to put forth their best efforts to achieve organizational goals; he or she also emphasizes developing the employees by improving their capabilities and recognizing their efforts on the other side. The early researchers have also established at many levels that corporate leaders are in a great position to influence different employees’ outcomes [154,155]. Thus, hospital employees under the supervision of TL are expected to develop an enhanced level of trust and respect, leading them to show more commitment and desire to go beyond expectations to support their organization. Essentially, healthcare employees face stressful situations regularly, which may result in a resource loss and increased burnout risks. Therefore, we are in line with the previous researchers that, in the presence of TL, employees feel less risk of burnout [14,59,60].

Our results also highlight the important mediating effects of role clarity and resilience between the relationship of TL and burnout in a hospital organization. Specifically, the statistical findings indicated both variables (role clarity and resilience) significantly explained the association between TL and burnout. Employees with clear role perceptions in a hospital are well aware of their tasks, which reduces the personal resource lost. Moreover, TL in a hospital is the one who provides clear direction and guidance to each employee about their roles and responsibilities. In other words, the presence of TL in a hospital ensures that employees are not off-tracked, especially while facing a stressful situation (which is a common norm in healthcare). When every employee has a clear role perception, as an outcome of TL, he or she is expected to face less risk of burnout. These lines of reasoning receive support from the early researchers too [66,67].

In a similar manner, the mediating role of resilience was also significant in the relationship between TL and burnout. In this respect, we are in line with the early work of Brennan [72], who believed that resilience is a psychological factor related to employees’ emotional well-being and professional success. Employees high in resilience are expected to find and use different resources (internal and external) to overcome the negative effects associated with burnout. On the part of employees, resilience enables them to deal with workplace stressors effectively by adapting to different workplace fluctuations. TL, in this respect, can serve as a critical factor that gives rise to employee resilience in a healthcare context. Such leaders help employees to reduce the risk of burnout on the one end, they execute strategies for the renewal of employees, including mindfulness practices and personal resilience plans on the other end, which makes employees more resourceful, leading them to a reduced level of burnout. 

Lastly, the empirical results also supported the conditional indirect effect of intrinsic motivation between the mediated relationship of TL and burnout through role clarity and resilience. In this respect, we are in line with Zhu, et al. [122], who suggested that an effective leader as a bottom-up approach induces the intrinsic motivation of employees, which then triggers different employee behaviors, including resilience. The results of this study suggest that intrinsic motivation of employees as an outcome of leadership may activate employee resilience. Further, a leader with a transformational approach displays an objective self-evaluation, appreciation for employees for their achievements, and maintains open communications with them. At the same time, TL focuses on employees’ welfare, enhancing their intrinsic motivation level, ultimately buffering their resilience and reducing employee burnout.

Additionally, the inspirational ability of TL focuses on enhancing the motivation level of employees by showing concern for fulfilling the needs and solving problems of employees. Thus, TL induces intrinsic motivations in employees. The seminal work by Brief and Aldag [156] indicated that intrinsically motivated employees do their job by showing an extra level of motivation due to feelings of self-fulfillment. In a healthcare context, intrinsically motivated employees work to serve humanity, reducing any role conflict and performing different tasks due to their inner feelings of serving humanity. This inner feeling improves their understanding of the complex nature of their job. This whole process enhances role clarity. All in all, in a hospital, an effective leader induces the intrinsic motivation of employees, which then produces a buffering effect between TL and role clarity, which ultimately buffers the negative effect of burnout.

### 5.1. Implications 

#### 5.1.1. Theoretical Implications

Our work extends the debate on burnout literature by providing the following insights on a theoretical landscape. First, our work is one of the limited studies that approaches TL from a perspective of burnout in a healthcare context. In this respect, the bulk of the existing literature investigated the positive aspects of TL [157,158]. Some recent investigations approached TL from a burnout perspective [14,19,60]; however, such studies are sparse, and most of them were not conducted in a healthcare context where employees face more burnout risks compared to other service contexts. Second, to our best knowledge, our research is the first one that advances the debate on burnout by incorporating the simultaneous effects of role clarity, resilience, and intrinsic motivation in a single unified model. Previous researchers investigated the mediating effect of resilience and role clarity on burnout [12,159]; such studies did not explain how resilience and role clarity simultaneously act as mediators to reduce the risk of burnout. More specifically, our research advances the theoretical framework by Djourova, et al. [92], who tested the mediating effect of resilience between TL and burnout but failed to consider the important role of role clarity and intrinsic motivation. Similarly, the conditional indirect role of intrinsic motivation on employee outcomes in a TL framework was highlighted by Shafi, et al. [160], but their study was conducted in a different context. Lastly, and most importantly, our research advances the burnout debate by highlighting its critical association with increasing medical errors in a developing country context (Pakistan). Given that hundreds of patients die in Pakistan on a daily basis due to medical errors, and considering the rising level of burnout in the healthcare sector, it was important to investigate how burnout in this sector can be reduced. Compared to developed countries, the phenomenon of burnout from a medical error perspective in a healthcare context was less studied in developing countries [7,161]. In this respect, healthcare employees face more difficult workplace situations than employees in developed countries because the healthcare systems in developing countries face more resource scarcity, which ultimately creates tough working and social conditions for healthcare employees [162]. Such differences, such as resource scarcity, insufficient structure, and the poor doctor-to-patient and nurse-to-patient ratios, require more investigations on the factors that can reduce employee burnout in developing countries’ healthcare systems. Therefore, our study enriches the available literature from a developing country context.

#### 5.1.2. Practical Implications

From a practical aspect, our research provides needful insight into the healthcare sector of Pakistan in dealing with the critical issue of burnout through TL. Considering the critical importance of burnout, our research presents a viable solution to deal with the employee burnout issue by converting the hospital managers into transformational leaders. The risk of burnout in a healthcare system undermines the quality of patient care by the hospital staff, which increases the chances of medical errors. However, the presence of TL in this respect may reduce employees’ burnout perceptions, which ultimately improves patients’ care and quality of service delivery. Similarly, the role of personal characteristics, such as intrinsic motivation and resilience, is also critical in dealing with the burnout risk in a TL framework. Hospital employees with a higher level of intrinsic motivation and resilience face less resource constraints, which reduce the chances for such employees to be emotionally drained while performing their job or facing a stressful situation. 

### 5.2. Limitations and Possible Future Directions

As with all survey research, our research also faces some potential issues, which may be called limitations. In this vein, the first limitation of our study rests with the geographical consideration, as the current study collected the data from two large cities. Though, these two cities were critical to investigate as these cities comprise a large number of hospitals. Still, we feel a better strategy could be to include more cities in order to have a better generalizability claim for this research. Therefore, for future studies, it is suggested to include more cities. A nonprobability sampling method was another potential limitation of this survey. Given that, due to different policy and safety issues, most hospitals did not share with us any list of employees, which could serve as a sampling frame to apply a probability sampling, we were unable to introduce any probability sampling technique. There is not any doubt in believing that probability sampling is regarded as superior compared to nonprobability sampling. Therefore, if possible, we suggest future studies subscribe to any probability sampling (for example, random sampling) method. Similarly, this study used conservation of resource theory to underpin the theoretical argument. In this respect, other related theories, for example, the demand-control-support model, may also be employed in future studies. Lastly, as other work stressors, such as role conflict and role overload, are also important predictors of burnout, we suggest future researchers include these variables in the current framework of this study.

## 6. Conclusions

Medical errors have been identified as one of the greatest evils in the field of healthcare, causing millions of patient deaths around the globe each year, especially in developing and poor countries. Different factors contribute to an increase in medical errors, including employee burnout. Taking corrective and preventive measures, it is expected that the criticality of medical errors can be improved in the healthcare sector. In this respect, we suggest the hospital management strongly think about fostering the transformational leadership style as a remedy to burnout risk. A stressful situation is a commonly observed phenomenon in healthcare that may give rise to the risk of burnout on the part of employees. However, a manager as a transformational leader helps employees to face less burnout situations by supporting, motivating, encouraging, and providing them with the necessary help to avoid resource depletion of employees. We, in this regard, suggest the management of the hospital arrange different training sessions, especially for the managers, with a special focus on highlighting the important benefit that a transformational leader can bring to the field. Further, we also suggest hospital management provide clear guidance on the roles, responsibilities, and tasks because employees with clear role perceptions are more resourceful and know what is to be performed by whom. Additionally, we also suggest redefining the employee screening criteria at the time of hiring. In this respect, some mechanics need to be applied to each candidate, which could indicate the intrinsic motivation and resilience-related attitude. To conclude, medical errors have existed in the field since the beginning and perhaps will exist in the future too, but, with different corrective steps, the severity of medical errors could be mitigated surely, for which the role of a transformational leader is of utmost importance.

## Figures and Tables

**Table 1:** Data cleaning, outliers, and response rate.

	Distributed	Returned	Unreturned	Unusable	Outliers	Final
	700	453	247	37	18	398
Percentage	-	64.71	35.29	5.286	2.571	56.86

**Table 2:** Socio-demographic information.

Demographic	Frequency (*n* = 398)	%
Gender		
Male	227	57.03
Female	171	42.96
Age		
18–25	47	11.81
26–30	59	14.82
31–35	102	25.62
36–40	74	18.59
41–45	70	17.59
Above 45	46	11.55
Experience		
1–3	82	20.60
4–6	124	31.15
7–9	113	28.40
Above 10	79	19.85

**Table 3:** Validity and reliability.

	Λ	λ^2^	S.E	T.Values	E-Variance	AVE	CR
TL						0.573	0.899
	0.728	0.530	0.072	10.11	0.470		
	0.713	0.508	0.075	09.51	0.492		
	0.803	0.645	0.066	12.17	0.355		
	0.757	0.573	0.070	10.81	0.427		
	0.819	0.671	0.064	12.80	0.329		
	0.707	0.500	0.076	09.30	0.500		
	0.764	0.584	0.069	11.07	0.416		
BO						0.605	0.914
	0.788	0.621	0.067	11.76	0.379		
	0.826	0.682	0.063	13.11	0.318		
	0.702	0.493	0.077	09.12	0.507		
	0.701	0.491	0.077	09.10	0.509		
	0.833	0.694	0.062	13.44	0.306		
	0.779	0.607	0.068	11.46	0.393		
	0.804	0.646	0.066	12.18	0.354		
RLC						0.608	0.903
	0.863	0.745	0.060	14.38	0.255		
	0.814	0.663	0.065	12.52	0.337		
	0.701	0.491	0.077	09.10	0.509		
	0.700	0.490	0.077	09.09	0.510		
	0.783	0.613	0.068	11.51	0.387		
	0.805	0.648	0.066	12.20	0.352		
RSL						0.571	0.889
	0.800	0.640	0.067	11.94	0.360		
	0.703	0.494	0.077	09.13	0.506		
	0.755	0.570	0.059	12.80	0.430		
	0.768	0.590	0.069	11.13	0.410		
	0.739	0.546	0.071	10.41	0.454		
	0.767	0.588	0.069	11.12	0.412		
IMO						0.642	0.889
	0.911	0.830	0.052	17.52	0.170		
	0.838	0.702	0.061	13.74	0.298		
	0.722	0.521	0.073	09.89	0.479		
	0.706	0.498	0.076	09.29	0.502		
	0.811	0.658	0.065	12.48	0.342		

Notes: λ = item loadings, CR = composite reliability, ∑λ^2^ = sum of square of item loadings, E-Variance = error variance.

**Table 4:** Model fit comparison, alternate vs. hypothesized models.

Model	Composition	*χ* ^2^	*df*	*χ^2^*/*df* (<3)	Δ*χ*^2^/*df* *-*	NFI (>0.9)	CFI (>0.9)	RMSEA (<0.08)
1	(hypothesized)	914	461	1.982	-	0.954	0.952	0.040
	TL, BO, RLC, RSL, IMO							
2	(3-factor)	2008	470	4.273	2.291	0.782	0.782	0.072
	TL + RLC + RSL, IMO, BO							
3	(2-factor)	2434	478	5.093	0.820	0.688	0.674	0.0910
	TL + RLC + RSL, IMO + BO							
4	(1-factor)	4016	480	8.366	3.273	0.511	0.532	0.102
	TL + RLC + RSL + IMO + BO							

**Table 5:** Correlations and discriminant validity.

Construct	TL	BO	RLC	RSL	IMO	Mean	SD
TL	0.757	−0.583	0.464	0.405	0.270	3.11	0.69
BO		0.778	−0.567	−0.485	−0.505	2.87	0.72
RLC			0.780	0.463	0.388	2.98	0.73
RSL				0.756	0.372	3.42	0.61
IMO					0.801	3.20	0.67

Notes: SD = standard deviation, diagonal = discriminant validity values.

**Table 6:** Direct, indirect, and conditional effects.

Hypotheses	Estimates (SE)	*t*/*z*	*p*-Value	CI
(TL→RLC)	0.4327 (0.0788)	05.4943	0.000	0.399, 0.533
(RLC→BO)	−0.3927 (0.0631)	−06.2272	0.006	−0.516, −0.268
(TL→RSL)	0.1251 (0.1251)	08.8727	0.002	0.339, 0.789
(RSL→BO)	−0.4912 (0.0692)	−07.0982	0.000	−0.394, −0.259
(TL→BO)	−0.3490 (0.0421)	−08.2897	0.000	−0.386, −0.303
Indirect effect	−0.1699 (0.0160)	−08.625	0.002	−0.172, −0.115
(TL→RLC→BO)				
(TL→RSL→BO)	−0.0614 (0.0101)	−10.200	0.000	−0.180, −0.091
Conditional indirect effect	−0.102 (0.0102)	−10.200	0.000	−0.180, −0.091
When RLC is a mediator				
Conditional indirect effect	−0.0382 (0.0130)	−2.938	0.007	−0.162, −0.086
When RSL is a mediator				

Notes: CI = 95% confidence interval with lower and upper limits.

## Data Availability

Data will be made available on a reasonable request by contacting the corresponding author(s).

## Appendix A

Transformational Leadership
Our leader communicates a clear and positive vision of the future
Our leader treats staff as individuals, supports and encourages their development
Our leader gives encouragement and recognition to staff
Our leader fosters trust, involvement and cooperation among team members
Our leader encourages thinking about problems in new ways and questions assumptions
Our leader is clear about his/her values and practices what he/she preaches
Our leader instills pride and respect in others and inspires me by being highly competent
Burnout
I feel worn out at the end of the working day
I feel my work is emotionally exhausting
I feel that every working hour is tiring for me
I am exhausted in the morning at the thought of another day at work
I don’t have enough energy for family and friends during my leisure time
My work frustrates me
I feel burnt out because of my work
Role Clarity
I have clear planned goals and objectives for my job
I know that I have divided my time properly
I know what my responsibilities are
I know exactly what is expected of me
I feel certain about how much authority I have on the job
Explanation is clear of what has to be done
Resilience
I tend to bounce back quickly after hard times
I have a hard time making it through stressful events
It does not take me long to recover from a stressful event
It is hard for me to snap back when something bad happens
I usually come through difficult times with little trouble
I tend to take a long time to get over set-backs in my life
Intrinsic Motivation
I enjoy finding solutions to complex problems
I enjoy coming up with new ideas for products
I enjoy engaging in analytical thinking
I enjoy creating new procedures for work tasks
I enjoy improving existing processes or products
